# Maternal PTSD following Exposure to the Wenchuan Earthquake Is Associated with Impaired Mental Development of Children

**DOI:** 10.1371/journal.pone.0168747

**Published:** 2017-04-03

**Authors:** Dongge Cai, Zhongliang Zhu, Hongli Sun, Yanhua Qi, Lanying Xing, Xiaogui Zhao, Qiuyuan Wan, Qian Su, Hui Li

**Affiliations:** 1 Department of Obstetrics and Gynecology, the First Affiliated Hospital of Xi’an Jiaotong University, Xi’an, Shaanxi, P. R. China; 2 Department of Obstetrics and Gynecology, the Second Affiliated Hospital of Xi’an Jiaotong University, Xi’an, Shaanxi, P. R. China; 3 Shaanxi Province Biomedicine Key Laboratory, College of Life Science, Northwest University, Xi’an, Shaanxi, P. R. China; 4 Department of Pharmacology, Xi'an Jiaotong University Health Science Center, Xi’an, Shaanxi, P. R. China; 5 Shaanxi Institute of Pediatric Diseases, Xi’an Children’s Hospital, Shaanxi, P.R. China; 6 Department of Ultrasound, the Second Affiliated Hospital of Xi’an Jiaotong University, Xi’an, Shaanxi, P. R. China; 7 Department of Neonatology, the First Affiliated Hospital of Xi’an Jiaotong University, Xi’an, Shaanxi, P. R. China; Stellenbosch University, SOUTH AFRICA

## Abstract

The purpose of this study was to explore whether earthquake-related maternal Post-Traumatic Stress Disorder (PTSD) is associated with impaired development of infants. Participants included 86 women who were pregnant during or after the earthquake in Ningqiang county, and their children. Data were collected from February to March of 2012. PTSD questionnaire (PTSD Checklist, Civilian Version (PCL-C)) was used to measure the effect of the earthquake on mothers, and that the scores greater than 50 were used to indicate presence of PTSD. Each child was assessed using the mental Developmental Screening Test (DST) according to age. Among the 86 women, PTSD scores equal to or greater than 50 accounted for 20.93%. Among the 86 children, 25.60% of development quotient (DQ) scores and 19.80% of mental index (MI) scores were less than 85. The correlation coefficient analysis showed that PTSD scores were inversely related to DQ and MI scores. Maternal PTSD following earthquake exposure is associated with relatively lower intellectual development in children age 0–3 years. Further research is needed to assess the persistent effects of this influence on offspring of mothers exposed to earthquake.

## Introduction

Fetal development is important to ensure the future health of an individual. Many studies on humans and experimental research with animals suggest that maternal stress during pregnancy, likely in interaction with genetic factors, may have long-lasting adverse consequences with respect to pregnancy outcomes [1,2] and the cognitive development and behavioral problems of offspring [3–6]. Such stress can result from natural or man-made disasters such as earthquakes, floods, ice storms, war, or terrorist acts. Previous studies have shown that mental health disorders such as post-traumatic stress disorder (PTSD) and other psychological health problems were common among earthquake survivors [7–12]. Furthermore, for similar catastrophic events that cause extraordinary destruction and disruption, populations may exhibit strikingly high levels of depression and post-traumatic stress for years after the disaster [13,14].

Several studies since the 1990s have found that women are disproportionately vulnerable to negative mental and physical health outcomes following disasters [15–17]. Arnberg et al. [18] measured symptoms of PTSD in 82 women 6 years after the 2004 Indian Ocean disaster, and found that the incidence of PTSD was still quite high among women who were on vacation in Southeast Asia during the disaster. Oni et al. [19] found that levels of PTSD and depression of pregnant women increased in relation to the severity of the experience of the hurricane.

An earthquake of magnitude 8.0 occurred in 2008 in Wenchuan County of Sichuan Province in southwest China. Over 270,000 pregnant women and 12 million women of child-bearing age were affected by the earthquake [20]. The aftermath of the earthquake was a source of enormous psychological stress, and the experiences of pregnant women may have influenced not only their own health but also that of their children.

The present study focused on whether earthquake-related maternal PTSD is associated with impaired development of infants. Specifically, PTSD symptoms among women who were pregnant during or after the earthquake and the impaired mental development of their infants were examined. A representative area close to the earthquake's epicenter, Ningqiang county, was most severely damaged and chosen as the setting for this study.

## Materials and Methods

### Study design and setting

The present work was conducted from February to March of 2012 (about 4 years after earthquake). Eighty-six women who were pregnant during or after the earthquake were recruited from the puerperants at the Ningqiang People's Hospital. Face-to-face interviews were conducted with participants at the Ningqiang People's Hospital. This study protocol was approved by the Institutional Review Board (IRB) of the First Affiliated Hospital of Xi’an Jiaotong University. A pediatrician, obstetricians, psychological experts, volunteers, and undergraduates who majored in pediatrics comprised two teams of investigators. Both teams received training by the authors of this study on communication and interviewing skills before conducting the interviews and data collection.

### Subjects and sampling methods

Detailed information and data about the damage caused by the earthquake were obtained during our investigation. A psychological assistant was present during the entire data collection session to answer queries from participants. Three obstetricians identified women who met inclusion criteria: (a) pregnant during the earthquake or became pregnant within 3 years after the earthquake, (b) Han nationality, (c) 18 years or older, (d) no other traumatic event during the investigation. Participation was discontinued for some participants because of obstetrical complications, such as premature births, stillbirths, and maternal medical complications (n = 14), and gemellary pregnancies (n = 3), leaving 86 mother-child pairs who continued to participate in the study. Only infants born after 37 weeks of gestation were included in the study. Written consent was obtained from participants. All women were paid 500 yuan for their time and effort.

### Variables

The following sociodemographic information was obtained for parents: 1) parent’s age (< 30 years or ≥ 30 years), 2) mother’s education level (< 9 years or ≥ 9 years), 3) living situation (village or city), 4) mother’s tobacco use (yes or no), and 5) alcohol use (yes or no). Among the participants, no one drank or smoked during pregnancy.

Pregnancy and child-related information was also obtained:

1) number of deliveries (primipara or multipara), 2) delivery type (normal delivery or Caesarean section), 3) child’s gender, 4) child’s birth weight, 5) child’s age.

### Measurements

#### Symptoms of post-traumatic stress disorder (PTSD)

We assessed the symptoms of PTSD using the PTSD Checklist, Civilian Version (PCL-C) compiled by Weathers et al. in 1993 [21]. This questionnaire is a self-assessment instrument widely used to measure traumatic stress relating to 17 PTSD-like symptoms. Using a 5-point scale, respondents indicate the degree to which they were bothered in the past four years by the most traumatic event of their life. The items of the PCL-C are divided into 3 sections: re-experiencing of trauma (Items 1–5), avoidance (Items 6–12), and hyperarousal (Items 13–17). Re-experiencing symptoms are intrusive thoughts about trauma, nightmares, flashbacks, distress, and physiological changes when reminded of trauma. Avoidance symptoms include avoidance of traumatic thoughts and reminders, lack of interest, detachment, emotional numbing, psychogenic amnesia, and sense of foreshortened future. Hyperarousal symptoms include insomnia, irritability, memory/concentration difficulties, hypervigilance, and startle response. A total score is obtained by adding the frequency and severity scores across all items (range = 15–85). A cutoff value of 50 was used to define a positive PTSD result; higher scores corresponded to greater symptoms of PTSD in the present study [22].

#### Child measures

The mental Developmental Screening Test (DST), developed by Children’s Hospital of Fudan University, remains the most widely used objective assessment of the level of mental development in Chinese infants [23]. The scale may be used with children from birth to age 6. It consists of 120 items, arranged in 3 categories: social adaptability, motor ability, and intelligence development. The DST quantitative indexes are expressed as the developmental quotient (DQ) and mental indexes (MI). The score of the DQ is the total of the three categories, which provides an integrated overview of pediatric development. MI measures the development of pediatric intelligence. The correlation coefficients for inter-observer and test-retest reliability were 0.94 and 0.90, respectively. Prior to test administration, the age of the infant is calculated as of the current date. For ease of interpretation, children were classified into 4 groups based on age: less than 1 year old, 1–2 years old; 2–3 years old, and 3–4 years old. Each child was tested in a quiet area according to age. The development quotient (DQ) and mental index (MI) were calculated according to the age of the children. A cutoff value of 85 was used to define a positive DST result in the present study, which means that DST scores less than 85 signify delay of social adaptability, motor ability, and intelligence development. If a child obtained a DST scores less than 85, the parent was informed, and a recommendation was made to obtain further diagnosis or treatment.

### Statistical methods

Statistical analysis was performed using SPSS 18.0. First, descriptive analyses were conducted on demographic characteristics (age, number of deliveries and type, living situation, education level), smoking and drinking behaviors, earthquake exposure indicators, and outcome variables (PTSD symptoms, DQ, and MI). Second, we tested correlations between the outcome and predictor variables and sociodemographic factors using Chi-square tests and t-tests. Third, Pearson correlation coefficient and Spearman rank correlation coefficient analysis were performed for each outcome variable. A p value less than 0.05 was considered to be statistically significant.

## Results

### Demographics and socioeconomic status

All 86 women were married and had received at least 6 years of education (mean = 10.1 years). The mean age of the 86 women was 29.3 years (range 18–40 years), and half were over 30 years old. Overall, 60.46% lived in villages and 39.53% lived in cities. No significant differences were observed in the proportions of low DQ and MI scores between the two maternal age groups (p > 0.05). Similarly, no significant differences were observed between the two age groups of fathers (p > 0.05). For DQ and MI scores, the proportions of scores less than 85 were not significantly different between mothers with more or less than 9 years of education (p > 0.05), and between mothers living in villages versus cities (p > 0.05) (Table 1).

**Table 1:** Chi-square statistics of socio-demographic, pregnancy-related and child-related characteristics for DQ and MI.

Variable	DQ score<85(n,%)	DQ score≥85(n,%)	P	MI score<85(n,%)	MI score≥85(n,%)	P
**Mean value**	78.45	113.14		83.91	117.84	
**Mother age**						
≤30y	10(11.63)	33(38.37)	0.62	9(10.47)	34(39.53)	0.79
>30y	12(13.95)	31(36.05)		8(9.30)	35(40.70)	
**Education degree**						
≤9years	8(9.30)	25(29.07)	0.82	11(12.79)	32(37.21)	0.18
>9years	14(16.28)	39(45.35)		6(6.98)	37(43.02)	
**Husband’s age**						
≤30years	10(11.63)	30(34.88)	0.91	8(9.30)	30(34.88)	0.80
>30years	12(13.95)	34(39.53)		9(10.47)	39(45.35)	
**Living situation**						
Village	13(15.12)	39(45.35)	0.88	11(12.79)	48(55.81)	0.70
City	9(10.47)	25(29.07)		6(6.98)	21(24.42)	
**Delivery times**						
primiparae	10(11.63)	34(39.53)	0.54	5(5.81)	18(20.93)	0.78
multiparae	12(13.95)	30(34.88)		12(13.95)	51(59.30)	
**Delivery type**						
Normal delivery	11(12.79)	23(26.74)	0.25	7(8.14)	31(36.05)	0.78
Caesarean section 11(12.79)		41(47.68)		10(11.63)	38(44.18)	
**Time of conceive**						
Before earthquake 3(3.49)		16 (18.61)	0.27	3(3.49)	16(18.61)	0.62
After earthquake	19(22.09)	48(55.81)		14(16.28)	53(61.62)	
**Trimester of pregnancy**						
First trimester	1(5.26)	4(21.06)	0.96	2(10.53)	3(15.79)	0.16
Second trimester	1(5.26)	6 (31.58)		0(0.00)	8(42.15)	
Third trimester	1(5.26)	6(31.58)		1(5.26)	5(26.33)	
**Children’s gender**						
Male	18(20.93)	36(41.86)	0.03	10(11.63)	44(51.16)	0.71
Female	4(4.65)	28(32.56)		7(8.14)	25(29.07)	
**Children’s birth weight**						
≤2500g	5(5.81)	31(36.05)	0.04	6(6.98)	24(27.91)	0.97
>2500g	17(19.77)	33(38.37)		11(12.79)	45(52.32)	
**Children’s age**						
0–1	3(3.49)	11(12.79)	0.23	2(2.33)	15(17.44)	0.37
1–2	7(8.14)	13(15.12)		4(4.65)	10(11.63)	
2–3	9(10.47)	29(20.93)		8(9.30)	28(32.56)	
3–4	3(3.49)	16(25.57)		3(3.49)	16(18.60)	

*Notes*: MI = mental index; DQ = development quotient.

### Impact of timing and stage of pregnancy on DQ and MI of children

The ratio of low DQ and MI scores in children whose mother was pregnant before the earthquake were both 15.79%, and that in children whose mother was pregnant after the earthquake was28.36% and 20.90%, respectively. The rates of DQ < 85 and MI < 85 were not significantly different between the two groups whose mothers were pregnant before or after the earthquake (p > 0.05). The results also showed that children whose mothers experienced the earthquake in different trimesters of pregnancy did not show different proportions of low DQ and MI scores (Table 1).

### Impact of delivery type on DQ and MI of children

We explored whether obstetric factors might have confounded the interpretation of findings with respect to the incidence of DQ < 85 and MI < 85. The proportions of low DQ and MI scores in the normal delivery group were 32.35% and 18.42%, respectively, and in the caesarean section group were 21.15% and 20.83%, respectively. The proportions of DQ < 85 and MI < 85 were not significantly different between delivery types (p > 0.05) (Table 1).

### DQ and MI by child age, gender, and birth weight

Among the 86 children, there were 54 boys and 32 girls. The mean DQ score was 98.59 ± 2.08, and 22 DQ scores (18 boys and 4 girls) were less than 85 (25.6%). The mean MI score was 97.35 ± 1.64, and 17 MI scores (10 boys and 7 girls) were less than 85 (19.8%). The incidence of DQ < 85 was 33.3% for boys, which was 12.50% higher than for girls (p < 0.05). The incidence of MI < 85 was 18.52% among boys, which was not significantly different from the proportion among girls (21.80%) (p > 0.05). The proportions of DQ < 85 and MI < 85 did not differ significantly by child age (p > 0.05). The proportions of DQ < 85 differ significantly by birth weight (p < 0.05) (Table 1).

### Posttraumatic stress disorder (PTSD)

Among the 86 women, the mean score on the PCL-C was 38.55 ± 1.56, and 20.93% of scores were equal to or greater than the cutoff of 50, defining PTSD. The incidence of DQ < 85 was 61.11% among children whose mothers’ PCL-C scores were above the cutoff, compared to 16.18% among children whose mothers’ scores were less than 50 (p < 0.01). However, the proportion of MI < 85 (27.78%) among children whose mothers’ PTSD scores were equal to or greater than 50 was not significantly different from the proportion for those with PTSD scores less than 50 (30.77%) (p > 0.05). Subsequent analyses showed that children whose mothers had higher PTSD scores had lower scores on both the DQ and MI (p < 0.01, Table 2)

**Table 2:** Chi-square statistics for PTSD, DQ, and MI scores

Variable	DQ^a^ score < 85 (n, %)	DQ score ≥ 85 (n, %)	p	MI^b^ score < 85 (n, %)	MI score ≥ 85 (n, %)	p
PTSD^c^ score						
≤50	11 (12.79)	57 (66.28)	<0.01	12 (13.95)	56 (65.12)	0.34
>50	11 (12.79)	7 (8.14)		5 (5.81)	13 (15.12)	

^a^Development quotient

^b^Mental index

^c^Post-traumatic stress disorder

### Correlations among maternal sociodemographic information, maternal -related and child-related variables, PTSD, DQ, and MI

The Pearson correlation coefficient and Spearman rank correlation coefficient analysis showed that PTSD was associated with mother’s age (r = 0.23, p < 0.05) such that older adults were at greater risk for developing posttraumatic sequelae than younger adults. PTSD scores were also inversely related to DQ and MI scores (r_1_ = -0.78, r_2_ = -0.54, p < 0.05). The results also showed that DQ and MI scores were associated with gender (r_1_ = -0.20, r_2_ = -0.23, p < 0.05), such that boys had obtained lower scores than girls. The results also suggested that DQ and MI scores were associated with trimester of pregnancy when the earthquake occurred, meaning that when the earthquake occurred, the later the trimester of the pregnancy, the higher the DQ and MI score (Table 3).

**Table 3:** Correlation coefficients between all outcome and predictor variables

Variable (RP)	1	2	3	4	5	6	7	8	9	10	11	12	13
**1 Mother’s Age**	-												
**2 Education**	-0.00	-											
**3 Father’s age**	0.03	0.01	-										
**4 Child’s gender**	-0.01	0.03	0.06	-									
**5 Child’s birth weight**	-0.01	0.00	0.01	0.04	-								
**6 Child’s age**	0.01	0.07	0.02	0.01	0.09	-							
**7 Delivery type**	-0.01	0.65*	0.03	0.08	0.03	0.02	-						
**8 Gestational age**	-0.01	0.01	0.03	0.01	0.03	0.08	0.02	-					
**9 PTSD^a^ score**	0.23*	0.09	0.07	0.09	0.04	0.07	0.01	0.08	-				
**10 DQ^b^**	-0.01	0.05	0.08	-0.20*	0.06	0.09	0.02	0.08	-0.78*	-			
**11 MI^c^**	-0.02	0.06	0.09	-0.23*	0.00	0.01	0.04	0.01	-0.54*	0.57*	-		
**12 Trimester of pregnancy**	0.09	0.12	0.14	-0.08	0.05	0.0854	0.09	0.01	0.02	0.68*	0.79*	-	
**13 Conception before earthquake**	0.03	-0.07	0.04	0.06	0.07	-0.04	0.05	0.00	0.10	0.08	-0.04	0.03	-

^a^Post-traumatic stress disorder

^b^Development quotient

^c^Mental index

*p < 0.05

## Discussion

PTSD is the most commonly studied and most frequent and serious psychological disorder that occurs after traumatic events or disasters [24]. In women of childbearing age, pregnancy may increase sensitivity to the impact of disaster and increase the risk of PTSD following severe trauma [25] or rekindle or exacerbate the disorder [26]. Goenjian et al. provided further evidence that those exposed to severe trauma, including earthquake trauma, had high PTSD scores that did not remit over the next 1–5 years [27]. Earthquakes allow us to study the effects of a single major psychological event that is randomly distributed across pregnant women. The prevalence of maternal PTSD was 20.93% four years after the earthquake, which was higher than PTSD rates recorded among women by Qu eighteen months after the earthquake (12.20%) and lower than the rate reported by Kuu 3 months after the earthquake (50.70%) [12,28]. In line with previous research, our findings suggested that older individuals were at greater risk for developing posttraumatic sequelae than younger adults; we also found that PTSD showed a significant correlation with age of the mother [29]. The reason for the difference between the observed rate of maternal PTSD in this study compared to other studies is unclear. However, it should be pointed out that geographical diversity, varying earthquake intensity, investigation period, sample selection procedures, and particularly participant age and different PTSD assessment methods across studies make it difficult to derive an estimate of the expected morbidity and risk factors of mental health consequences following earthquake disasters. Many previous studies found that other psychological disorders and their symptoms, such as depression, would arise along with PTSD and interact with each other among pregnant women and new mothers after natural or manmade disasters [10,30,31].

A work had reported on the effects of prenatal maternal stress caused by natural and man-made disasters on cognitive development, as well as language and motor abilities of their children. In a major study of women in Quebec, Canada who were pregnant during a major ice storm, greater prenatal stress of the mother predicted higher Autism Spectrum Screening Questionnaire scores in 6½-year-old children independent of potential confounds [32]. A similar study of the same ice storm clearly indicated that high levels of objective hardship and/or subjective prenatal maternal stress were associated with lower motor scores in children [33]. This study investigated the impact of the Ningqiang earthquake on the mental development of children born after the earthquake, over a period of 4 years. The data suggested that children whose mothers conceived before or after earthquake had lower scores on the DQ and MI. Likewise, those exposed to flooding or tornadoes also show similar effects, for example, somewhat lower math scores [34]. Although genetics and stress in the postpartum period clearly affect these outcomes for offspring, evidence for an additional prenatal causal component is substantial, based on studies of cross-fostered animals and children born after in-vitro fertilization [35,36], which establish that prenatal stress can have a direct effect on child development outcomes. However, not all studies have demonstrated such an association, and among those that have, not all have reported that mothers’ negative psychological states are related to pregnancy outcomes or children’s development [37,38]. Sometimes, results show a counter-intuitive relation between maternal stress and cognitive outcomes of infants [39,40]. Thus, based on these contradictory findings, further investigation of the specific characteristics of stressors that either accelerate or impair children's development is needed.

We reported a significant association between maternal PTSD after the earthquake and low DQ and MI scores in children. A study of women exposed to the World Trade Center attacks on 11 September 2001 and their fetuses indicated that post-traumatic stress symptomatology in mothers was associated with longer gestational periods and decrements in infant head circumference at birth [41]. Among previous studies, inconsistent and even controversial relationships have been reported between PTSD and children's development. The “Project Ice Storm” study found that children born to Quebec women who were pregnant during the major ice storm had poorer intellectual and language development at 2 years of age and these adverse effects were shown to persist until the child was 5.5 years old; however, the outcome had no association with subjective stress such as PTSD [42,43]. Reasons for these varying and sometimes contradictory stress outcomes are only just starting to be uncovered. Retrospective assessments of stress, potential covariates of prenatal stress, use of different instruments for assessing pregnant women and children, disaster types, trimester of exposure, intensity, and other biological factors could be potential factors.

The present study showed that the earthquake occurring during different trimesters of gestation did not have a different influence on children’s mental development. This result is different from previous research about childhood schizophrenia, which showed fetal sensitivity during early pregnancy [44, 45]. Previous research with adults suggested that being exposed to stress as a fetus because of famine during the first trimester of pregnancy was related to poor performance in terms of selective attention and cognitive ability, and self-reported mild-to-severe anxiety as an adult [46, 47]. Another study of women exposed to the Arab-Israeli war of 1967 indicated that the incidence of schizophrenia in their offspring who were in the middle term of fetal life was increased [48]. Other research has shown that persons exposed to stress during the second trimester of pregnancy are vulnerable to becoming mentally ill [49,50]. In present work, we found an association between DQ and MI scores and the trimester of pregnancy. This correlation suggested a trend, such that the earlier trimester of the pregnancy, the greater the negative impact was on children's mental development.

Another potential concern is the possibility that exposure to a disaster affects child development differently by gender. A previous study found that among children with prenatal exposure to the ice storm, girls performed better than boys with respect to motor development. However, boys’ performance did not change as a function of timing of the exposure, while girls’ performance became poorer as the exposure to stress was later in the pregnancy [33]. Some studies aimed at adults suggested that men who suffered from famine as fetuses during the first trimester scored lower on conscientiousness and higher on depression and anxiety. In contrast, women who suffered stress during the same fetal period scored higher on agreeableness [46,51]. Similar results were also found in research on the relationship between prenatal maternal stress and affective disorders [49,50]. These findings are similar to those from studies conducted with animals, which indicate that prenatal stress increases anxiety and depressive behavior to a greater extent in female offspring, while it leads to poor learning and cognitive development [52], and decreased learning capability and memory retrieval more in males [53]. The present findings indicated that lower DQ scores and incidence among boys were statistically higher than those among girls. The Pearson correlation coefficient analysis showed that DQ and MI scores were associated with gender, such that boys had a greater proportion of scores below the cutoff than girls.

It is necessary to note, however, that some study limitations exist. In particular, the sample size of our study was relatively small, which limited statistical power to detect statistical significance in associations, and limits generalizability to the general population. Moreover, assessments of stressors were carried out nearly 4 years after the earthquake, and problems with recollection of the events, such as life difficulties, may have reduced the stability of women's responses. Finally, the current study did not account for other emotional disorders, such as depression, which may be linked to children’s development outcomes.

However, although such limitations existed, the strength of the present study was the use of reliable and effective screening instruments such as the DST. The DST has a lower misdetection rate (8%) than that of the Denver Developmental Screening Test (50%) [54], and assesses children’s mental development comprehensively and continuously. In summary, this study conducted several levels of analyses and identified associations between maternal prenatal stress related to an earthquake and poor childhood mental development. We suspect that mothers suffering from PTSD may have children with disturbed fetal neurodevelopment, thereby influencing their neurobehavioral capacities in early childhood, and perhaps persisting throughout adulthood.

The data from the present study indicated different impacts of maternal stress on boys and girls, suggesting the importance of the investigation of sex-specific patterns of sensitivity to prenatal stress and their potential biological mechanisms. For the purpose of reducing poor mental development outcomes among children during and after severe disaster events, follow-up studies on this topic should also focus on the minimization of pregnant women’s exposure to negative events and the long-term effects of earthquake disasters on children.

## References

[1] Torche F, Kleinhaus K. Prenatal stress, gestational age and secondary sex ratio: the sex-specific effects of exposure to a natural disaster in early pregnancy. Human Reprod. 2012;27: 558–567. 10.1093/humrep/der390 PMC3258031 22157912

[2] Torche F. The effect of maternal stress on birth outcomes: exploiting a natural experiment. Demography. 2011;48: 1473–1491. 10.1007/s13524-011-0054-z 21870187

[3] O’Donnell K, O’Connor TG, Glover V. Prenatal stress and neurodevelopment of the child: focus on the HPA axis and role of the placenta. Dev Neurosci. 2009;31: 285–292. 10.1159/000216539 19546565

[4] Morley-Fletcher S, Rea M, Maccari S, Laviola G. Environmental enrichment during adolescence reverses the effects of prenatal stress on play behaviour and HPA axis reactivity in rats. Eur J Neurosci. 2003;18: 3367–3374. 14686910 10.1111/j.1460-9568.2003.03070.x

[5] Morley-Fletcher S, Mairesse J, Soumier A, Banasr M, Fagioli F, Gabriel C, et al Chronic agomelatine treatment corrects behavioral, cellular, and biochemical abnormalities induced by prenatal stress in rats. Psychopharmacology. 2011; 217: 301–313. 10.1007/s00213-011-2280-x 21503609

[6] Wilson CA, Vazdarjanova A, Terry AV Jr. Exposure to variable prenatal stress in rats: effects on anxiety-related behaviors, innate and contextual fear, and fear extinction. Behav Brain Res. 2013;238: 279–288. 10.1016/j.bbr.2012.10.003 23072929 PMC3534758

[7] Ehring T, Razik S, Emmelkamp PM. Prevalence and predictors of posttraumatic stress disorder, anxiety, depression, and burnout in Pakistani earthquake recovery workers. Psychiatry Res. 2011;185: 161–166. 10.1016/j.psychres.2009.10.018 20537401

[8] Fan F, Zhang Y, Yang Y, Mo L, Liu X. Symptoms of posttraumatic stress disorder, depression, and anxiety among adolescents following the 2008 Wenchuan earthquake in China. J Trauma Stress. 2011;24: 44–53. 10.1002/jts.20599 21351164

[9] Ren Z, Deng H, Hsu LK. PTSD in a one year old girl after the Wenchuan earthquake in Sichuan, China. Psychiatry. 2011;74: 87–92. 10.1521/psyc.2011.74.1.87 21463173

[10] Salcioglu E, Basoglu M, Livanou M. Post-traumatic stress disorder and comorbid depression among survivors of the 1999 earthquake in Turkey. Disasters. 2007;31: 115–129. 10.1111/j.1467-7717.2007.01000.x 17461919

[11] Varela E, Koustouki V, Davos CH, Eleni K. Psychological consequences among adults following the 1999 earthquake in Athens, Greece. Disasters. 2008;32: 280–291. 10.1111/j.1467-7717.2008.01039.x 18380855

[12] Qu Z, Tian D, Zhang Q, Wang X, He H, Zhang X, et al The impact of the catastrophic earthquake in China's Sichuan province on the mental health of pregnant women. J Affect Disord. 2012;136: 117–123. 10.1016/j.jad.2011.08.021 21937121

[13] Norris FH, Perilla JL, Riad JK, Kaniasty K, Lavizzo EA. Stability and change in stress, resources, and psychological distress following natural disaster: Findings from hurricane Andrew. Anxiety Stress Coping. 1999;12: 363–396. 10.1080/10615809908249317 21777067

[14] Brackbill RM, Cone JE, Farfel MR, Stellman SD. Chronic physical health consequences of being injured during the terrorist attacks on World Trade Center on September 11, 2001. Am J Epidemiol. 2014;179: 1076–1085. 10.1093/aje/kwu022 24561992 PMC4047283

[15] Fothergill A. Gender, risk, and disaster. International Journal of Mass Emergencies and Disasters. 1996;14: 33–56.

[16] Fothergill A. Heads above water: Gender, class, and family in the Grand Forks Flood. New York: SUNY Press; 2004.

[17] Kuo HW, Wu SJ, Ma TC, Chiu MC, Chou SY. Posttraumatic symptoms were worst among quake victims with injuries following the Chi-chi quake in Taiwan. J Psychosom Res. 2007;62: 495–500. 10.1016/j.jpsychores.2004.11.012 17383502

[18] Arnberg FK, Johannesson KB, Michel PO. Prevalence and duration of PTSD in survivors 6 years after a natural disaster. J Anxiety Disord. 2013;27: 347–352. 10.1016/j.janxdis.2013.03.011 23660149

[19] Oni O, Harville EW, Xiong X, Buekens P. Impact of coping styles on post-traumatic stress disorder and depressive symptoms among pregnant women exposed to Hurricane Katrina. Am J Disaster Med. 2012;7: 199–209. 23140063 10.5055/ajdm.2012.0095

[20] Liu SA, Zhou H, Zhou XF, Hu JF, Chen MT, Hu WJ, et al [Mental health assessment among scattered residents after Wenchuan earthquake in Anxian, Sichuan province]. Zhonghua Yu Fang Yi Xue Za Zhi [Chinese Journal of Preventive Medicine]. 2009;43: 380–384. 19534990

[21] Weathers FW, Litz BT, Herman DS, Huska JA, Keane TM. The PTSD Checklist (PCL): Reliability, validity, and diagnostic utility. 1993. Paper presented at the 9th Annual Conference of the International Society for Traumatic Stress Studies, San Antonio, TX.

[22] Blanchard EB, Jones-Alexander J, Buckley TC, Forneris CA. Psychometric properties of the PTSD Checklist (PCL). Behav Res Ther. 1996;34: 669–673. 8870294 10.1016/0005-7967(96)00033-2

[23] Zheng M, Feng L, Liu X. Standardization of the mental developmental screening test (DST) for children aged 0–6 years in China [In Chinese]. Chinese Journal of Pediatrics. 1997;35(3): 117–117.

[24] Galea S, Nandi A, Vlahov D. The epidemiology of post-traumatic stress disorder after disasters. Epidemiol Rev. 2005;27: 78–91. 10.1093/epirev/mxi003 15958429

[25] Thompson MP, Kingree J. The frequency and impact of violent traumaAmong pregnant substance abusers. Addict Behav. 1998;23: 257–262. 9573429 10.1016/s0306-4603(97)00032-4

[26] Josephs L. Women and trauma: a contemporary psychodynamic approach to traumatization for patients in the OB/GYN psychological consultation clinic. Bull Menninger Clin. 1995;60: 22–38. 8742670

[27] Goenjian AK, Steinberg AM, Najarian LM, Fairbanks LA, Tashjian M, Pynoos RS. Prospective study of posttraumatic stress, anxiety, and depressive reactions after earthquake and political violence. Am J Psychiatry. 2000;157: 911–916. 10.1176/appi.ajp.157.6.911 10831470

[28] Kun P, Chen X, Han S, Gong X, Chen M, Zhang W, et al Prevalence of post-traumatic stress disorder in Sichuan Province, China after the 2008 Wenchuan earthquake. Public Health. 2009;123: 703–707. 10.1016/j.puhe.2009.09.017 19892379

[29] Friedsam HJ. Reactions of older persons to disaster-caused losses: An hypothesis of relative deprivation. The Gerontologist. 1961;1: 34–37.

[30] Xiong X, Harville EW, Mattison DR, Elkind-Hirsch K, Pridjian G, Buekens P. Hurricane Katrina experience and the risk of post-traumatic stress disorder and depression among pregnant women. Am J Disaster Med. 2010;5: 181–187. 20701175 10.5055/ajdm.2010.0020 PMC3501144

[31] Qu Z, Wang X, Tian D, Zhao Y, Zhang Q, He H, et al Posttraumatic stress disorder and depression among new mothers at 8 months later of the 2008 Sichuan earthquake in China. Arch Womens Ment Health. 2012;15: 49–55. 10.1007/s00737-011-0255-x 22249399

[32] Walder DJ, Laplante DP, Sousa-Pires A, Veru F, Brunet A, King S. Prenatal maternal stress predicts autism traits in 6½ year-old children: Project Ice Storm. Psychiatry Res. 2014;219: 353–360. 10.1016/j.psychres.2014.04.034 24907222

[33] Cao X, Laplante DP, Brunet A, Ciampi A, King S. Prenatal maternal stress affects motor function in 5½-year-old children: Project Ice Storm. Dev Psychobiol. 2014;56: 117–125. 10.1002/dev.21085 23143986

[34] Fuller SC. The effect of prenatal natural disaster exposure on school outcomes. Demography. 2014;51: 1501–1525. 10.1007/s13524-014-0310-0 24903841

[35] Rice F, Harold GT, Boivin J, van den Bree M, Hay DF, Thapar A. The links between prenatal stress and offspring development and psychopathology: disentangling environmental and inherited influences. Psychol Med. 2010;12: 335–345. 10.1017/S0033291709005911 PMC2830085 19476689

[36] Afadlal S, Polaboon N, Surakul P, Govitrapong P, Jutapakdeegul N. Prenatal stress alters presynaptic marker proteins in the hippocampus of rat pups. Neurosci Lett. 2010;470: 24–27. 10.1016/j.neulet.2009.12.046 20035832

[37] Keim SA, Daniels JL, Dole N, Herring AH, Siega-Riz AM, Scheidt PC. A prospective study of maternal anxiety, perceived stress, and depressive symptoms in relation to infant cognitive development. Early Hum Dev. 2011;87: 373–380. 10.1016/j.earlhumdev.2011.02.004 21420261 PMC3079050

[38] Currie J, Rossin-Slater M. Weathering the storm: hurricanes and birth outcomes. J Health Econ. 2013;32: 487–503. 10.1016/j.jhealeco.2013.01.004 23500506 PMC3649867

[39] Davis EP, Sandman CA. The timing of prenatal exposure to maternal cortisol and psychosocial stress is associated with human infant cognitive development. Child Dev. 2010;81: 131–148. 10.1111/j.1467-8624.2009.01385.x 20331658 PMC2846100

[40] DiPietro JA. The role of prenatal maternal stress in child development. Curr Dir Psychol Sci. 2004;13: 71–74.

[41] Engel SM, Berkowitz GS, Wolff MS, Yehuda R. Psychological trauma associated with the World Trade Center attacks and its effect on pregnancy outcome. Paediatr Perinat Epidemiol. 2005;19: 334–341. 10.1111/j.1365-3016.2005.00676.x 16115284

[42] Laplante DP, Barr RG, Brunet A, Galbaud du Fort G, Meaney ML, Saucier JF, et al Stress during pregnancy affects general intellectual and language functioning in human toddlers. Pediatr Res. 2004;56: 400–410. 10.1203/01.PDR.0000136281.34035.44 15240860

[43] Laplante DP, Brunet A, Schmitz N, Ciampi A, King S. Project Ice Storm: prenatal maternal stress affects cognitive and linguistic functioning in 5½-year-old children. J Am Acad Child Adolesc Psychiatry. 2008;47: 1063–1072. 10.1097/CHI.0b013e31817eec80 18665002

[44] Khashan AS, Abel KM, Mcnamee R, Pedersen MG, Webb RT, Baker PN, et al Higher risk of offspring schizophrenia following antenatal maternal exposure to severe adverse life events. Arch Gen Psychiatry. 2008;65: 146–152. 10.1001/archgenpsychiatry.2007.20 18250252

[45] Susser E, Lin S. Schizophrenia after prenatal exposure to the Dutch Hunger Winter of 1944–1945. Arch Gen Psychiatry. 1992;49: 983–988. 1449385 10.1001/archpsyc.1992.01820120071010

[46] de Rooij SR, Painter RC, Phillips DI, Räikkönen K, Schene AH, Roseboom TJ. Self-reported depression and anxiety after prenatal famine exposure: mediation by cardio-metabolic pathology? J Dev Orig Health Dis. 2011;2: 136–143. 10.1017/S2040174411000055 25141038

[47] Rooij SRD, Wouters H, Yonker JE, Painter RC, Roseboom TJ. Prenatal undernutrition and cognitive function in late adulthood. Proc Natl Acad Sci U S A. 2010;107: 16881–16886. 10.1073/pnas.1009459107 20837515 PMC2947913

[48] Malaspina D, Corcoran C, Kleinhaus KR, Perrin MC, Fennig S, Nahon D, et al Acute maternal stress in pregnancy and schizophrenia in offspring: a cohort prospective study. BMC Psychiatry. 2008;8: 1–9. 18717990 10.1186/1471-244X-8-71 PMC2546388

[49] Machón RA, Mednick SA, Huttunen MO. Adult major affective disorder after prenatal exposure to an influenza epidemic. Arch Gen Psychiatry. 1997;54: 322–328. 9107148 10.1001/archpsyc.1997.01830160040006

[50] Brown AS, Susser ES, Lin SP, Neugebauer R, Gorman JM. Increased risk of affective disorders in males after second trimester prenatal exposure to the Dutch hunger winter of 1944–45. Br J Psychiatry. 1995;166: 601–606. 7620744 10.1192/bjp.166.5.601

[51] Rooij SRD, Veenendaal MVE, Räikkönen K, Roseboom TJ. Personality and stress appraisal in adults prenatally exposed to the Dutch famine. Early Hum Dev. 2012;88: 321–325. 10.1016/j.earlhumdev.2011.09.002 21955503

[52] Weinstock M. Gender differences in the effects of prenatal stress on brain development and behaviour. Neurochem Res. 2007;32: 1730–1740. 10.1007/s11064-007-9339-4 17406975

[53] Modir F, Salmani ME, Goudarzi I, Lashkarboluki T, Abrari K. Prenatal stress decreases spatial learning and memory retrieval of the adult male offspring of rats. Physiol Behav. 2014;129: 104–109. 10.1016/j.physbeh.2014.02.040 24582675

[54] Meisels SJ. (1988) Developmental screening in early childhood: the interaction of research and social policy. Annu Rev Public Health. 1988;9: 527–550. 10.1146/annurev.pu.09.050188.002523 2454121

